# Invasive Pneumococcal Disease Presenting as Austrian Syndrome in a Patient With HIV Infection: A Case Report

**DOI:** 10.7759/cureus.97768

**Published:** 2025-11-25

**Authors:** Shivani Jani, Misbahuddin Khaja

**Affiliations:** 1 Internal Medicine, BronxCare Health System, Bronx, USA; 2 Pulmonary and Critical Care Medicine, BronxCare Health System, Bronx, USA

**Keywords:** austrian syndrome, case report, clinical triad, infective endocarditis, meningitis, osler's triad, pneumococcal infection, pneumonia, streptococcus pneumoniae, tricuspid endocarditis

## Abstract

Austrian syndrome is a rare and severe manifestation of invasive *Streptococcus pneumoniae *infection, defined by the triad of pneumonia, meningitis, and infective endocarditis. Although it is uncommon in the modern antibiotic and vaccination era, Austrian syndrome is associated with significant morbidity and mortality. Here we present a case of a 62-year-old male with a history of human immunodeficiency virus infection and intravenous drug use who presented with cough, fatigue, and left upper extremity weakness. He developed acute hypoxic respiratory failure and status epilepticus requiring intubation. Workup revealed bibasilar pneumonia, *S. pneumoniae *bacteremia, and pneumococcal meningitis confirmed via cerebrospinal fluid analysis. Echocardiography demonstrated tricuspid valve vegetation consistent with endocarditis. The patient was managed with broad-spectrum intravenous antibiotics, which were subsequently de-escalated based on culture sensitivity. Surgical intervention was not opted for due to the size and location of the vegetations. The patient completed a six-week course of ceftriaxone and vancomycin with clinical improvement and negative follow-up blood cultures. This case explains the importance of maintaining a high index of suspicion for Austrian syndrome in high-risk populations presenting with systemic symptoms. Early recognition, microbiological and radiological confirmation, and aggressive antibiotic therapy are essential for a favorable outcome. Despite its rarity, awareness of this condition and adherence to evidence-based management can significantly improve survival in affected patients.

## Introduction

Austrian syndrome is characterized by a triad of pneumonia, meningitis, and infective endocarditis, most commonly caused by *Streptococcus pneumoniae*. It was first described by Robert Austrian in 1957, following autopsy results in patients with invasive pneumococcal infections [1]. Although each triad component can occur independently, their concurrent presence is relatively uncommon and is associated with higher morbidity and mortality. This syndrome is typically seen in patients with underlying risk factors such as chronic alcoholism, diabetes mellitus, asplenia, immunosuppression, and advanced age, predisposing them to invasive pneumococcal disease [2,3]. Among these, alcoholism has been frequently associated with Austrian syndrome [4]. While the incidence of invasive pneumococcal infections has markedly decreased due to the widespread use of pneumococcal conjugate vaccines, this condition remains a medical emergency due to its fulminant course and diagnostic complexity [5]. The diagnosis is delayed as the clinical presentation is usually dominated by pneumonia or meningitis, while endocarditis may be clinically silent until complications such as embolic phenomena or valvular dysfunction arise [6]. The aortic valve is the most frequently involved site in pneumococcal endocarditis, causing acute valvular insufficiency and rapid deterioration [7]. Austrian syndrome requires prompt identification and aggressive management with intravenous antibiotics and surgical intervention when indicated for favorable outcomes [8].

## Case presentation

A 62-year-old male with a medical history of asthma, stroke, human immunodeficiency virus (HIV) infection, and intravenous drug use presented to the emergency department with a cough with productive sputum, fatigue, and left upper extremity weakness for two days. Upon arrival, vital signs showed a temperature of 100.4°F, a blood pressure of 129/90 mmHg, a heart rate of 94 beats per minute, a respiratory rate of 18 breaths per minute, and an oxygen saturation of 94% on room air. The patient was noted to be in acute hypoxic respiratory failure requiring supplemental oxygen with a 2 L nasal cannula. Physical examination was significant for a systolic murmur at the left sternal border and left upper extremity weakness. Initial labs were significant for neutrophilia, thrombocytopenia, elevated N-terminal pro-brain natriuretic peptide, and elevated inflammatory markers such as C-reactive protein and lactate dehydrogenase (Table 1).

**Table 1:** Summary of hematological and biochemical workup.

Parameters	Day 1	Day 5	Day 10	Reference range
Hemoglobin	12.9 g/dL	11.7 g/dL	11 g/dL	12-16 g/dL
White blood cell count	6.9 k/μL	6.6 k/μL	15.5 k/μL	4.8-10.8 k/μL
Neutrophils %	85.7%	86.1%	92.2%	40-70%
Platelet	135 k/μL	197 k/μL	188 k/μL	150-400 k/μL
Sodium	136 meq/L	144 meq/L	147 meq/L	135-145 meq/L
Potassium	4.8	3.4	3.5	3.5-5
CO_2_	24 meq/L	22 meq/L	22 meq/L	24-30 meq/L
Blood urea nitrogen (BUN)	29 mg/dL	18 mg/dL	13 mg/dL	8-26.0 mg/dL
Creatinine	0.9 mg/dL	0.6 mg/dL	0.6 mg/dL	0.5-1.5 mg/dL
Calcium	8.7 mg/dL	8.3 mg/dL	7.9 mg/dL	8.5-10.5 mg/dL
Alanine aminotransferase (ALT)	17 U/L	12 U/L	23 U/L	5-40 U/L
Aspartate aminotransferase (AST)	40 U/L	20 U/L	54 U/L	9-48 U/L
Alkaline phosphatase (ALP)	64 U/L	47 U/L	62 U/L	56-155 U/L
Total bilirubin	0.7 mg/dL	0.3 mg/dL	0.4 mg/dL	0.2-1.1 mg/dL
Lactate dehydrogenase (LDH)	665 U/L	-	-	110-210 U/L
C-reactive protein	216 mg/L	-	-	<5 mg/L
Ferritin	632 ng/mL	-	-	13-150 ng/mL
Lactic acid	0.9 mmol/L	-	-	0.5-1.6 mmol/L
Pro-brain natriuretic peptide	285 pg/mL	510 pg/mL	-	0-125 pg/mL
Blood gas measurements				
pH	7.402	-	7.46	7.35-7.45
Partial pressure of carbon dioxide	46.5 mmHg	-	34.6 mmHg	35-45 mmHg
Partial pressure of oxygen	22.4 mmHg (venous)	-	188 mmHg	83-108 mmHg

**Figure 1:**
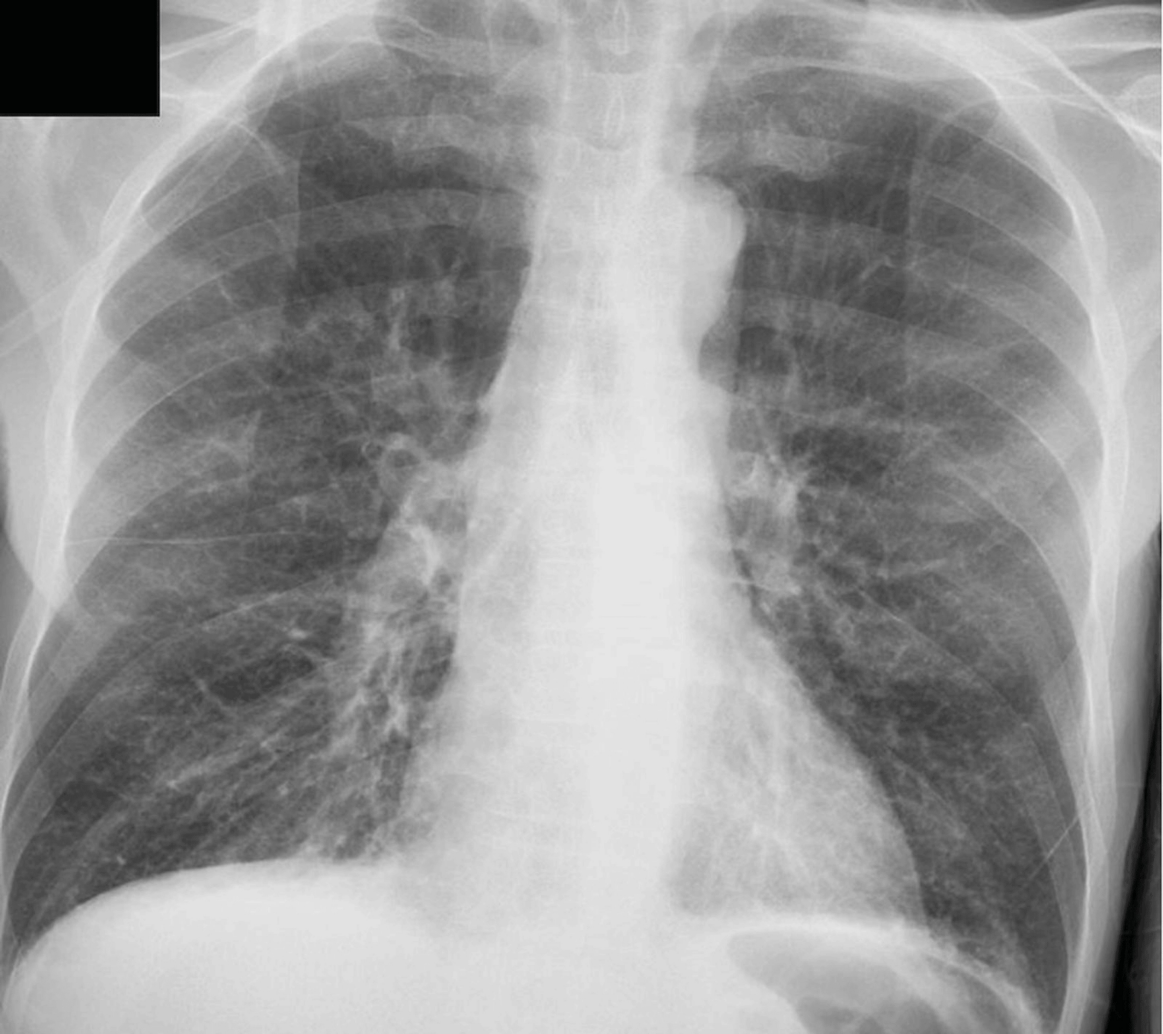
Chest X-ray showing pulmonary hyperinflation and pulmonary emphysema.

**Figure 2:**
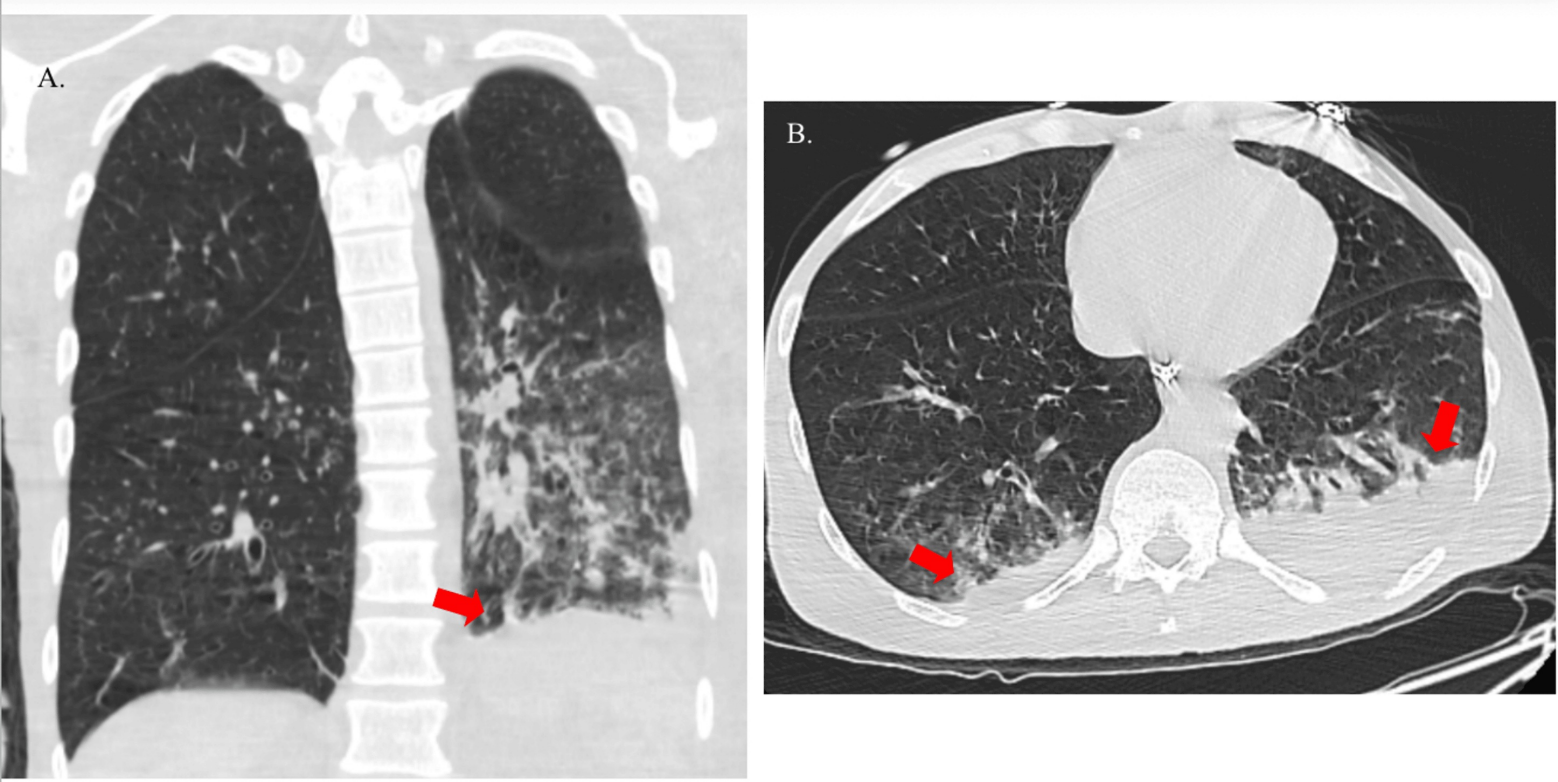
CT chest without contrast showing bibasilar consolidations (arrows). The images show (A) coronal view and (B) axial view.

The electrocardiogram was unremarkable. Chest X-ray reported pulmonary emphysema (Figure 1). Computed tomography of the brain was negative for acute events, and computed tomography angiography reported no significant stenosis of the carotid or vertebral arteries in the head and neck. Computed tomography of the chest showed bibasilar small consolidations, right larger than left, and a trace right pleural effusion (Figures 2A, 2B).

The patient was initially admitted for a suspected cerebrovascular accident and multifocal pneumonia. He was empirically started on ceftriaxone and azithromycin. The patient received highly active antiretroviral therapy with bictegravir/emtricitabine/tenofovir alafenamide, and *Pneumocystis jirovecii *prophylaxis with trimethoprim/sulfamethoxazole was initiated due to a low CD4 count of 48 cells/μL. The patient subsequently developed seizures complicated by status epilepticus, requiring intubation for airway protection. His antibiotic regimen was escalated to intravenous ceftriaxone, vancomycin, ampicillin, and acyclovir for empiric meningitis treatment. Lumbar puncture was performed, cerebrospinal fluid analysis showed neutrophilic leukocytosis, elevated protein, low glucose, and positive *Streptococcus pneumoniae *antigen by PCR. Two sets of blood cultures were positive for *Streptococcus pneumoniae*, susceptible to vancomycin. Nasopharyngeal swab for methicillin-resistant *Staphylococcus aureus *was also positive (Table 2).

**Table 2:** Cerebrospinal fluid analysis and microbiological studies. LDH: lactate dehydrogenase; MRSA: methicillin-resistant *Staphylococcus aureus*

Parameters	Results	Reference range
CSF analysis		
WBC (CSF)	12375 cells/μL	-
RBC (CSF)	2750 cells/μL	-
Protein (CSF)	761 mg/dL	15-45 mg/dL
Glucose (CSF)	<2 mg/dL	40-70 mg/dL
LDH (CSF)	554 U/L	-
Gram stain	Gram-positive cocci	-
CSF bacterial antigen	Streptococcus pneumoniae	-
CSF aerobic culture	No growth	-
Cryptococcal antigen	Not detected	Not detected
Mycobacterial culture	No growth	No growth
Other		
Respiratory viral panel	Negative	Negative
Blood culture	Streptococcus pneumoniae	No growth
*Streptococcus pneumoniae *antigen (urine)	Detected	Not detected
Fungal blood culture	No growth	No growth
Respiratory culture	No growth	No growth
MRSA PCR	Detected	Not detected
Urine culture	No growth	No growth
CD4 count	32 cells/μL	490-2740 cells/μL
HIV viral load	14600 copies/mL	Target not detected

**Figure 3:**
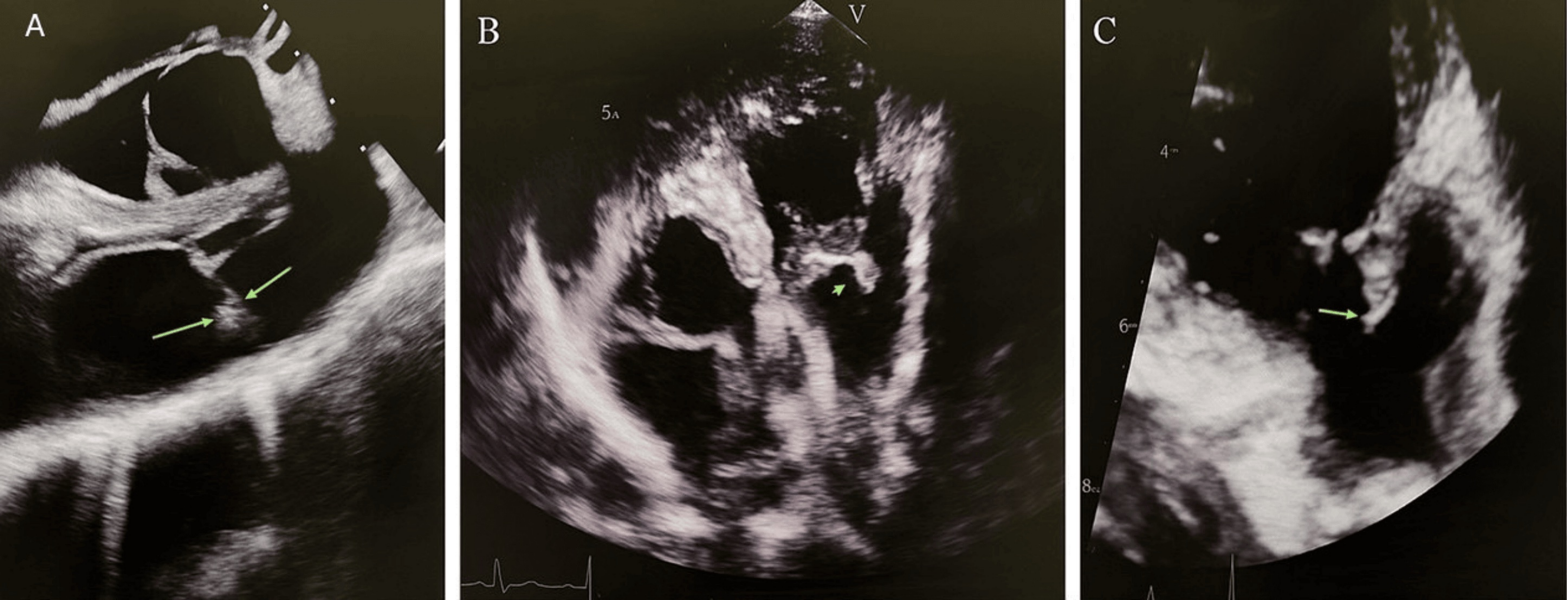
TEE showing a large, mobile, echodense structure measuring 0.7×1.22 cm attached to the anterior leaflet of the tricuspid valve, consistent with tricuspid vegetation (green arrows). The images show (A) parasternal long-axis view, (B) apical four-chamber view, and (C) transesophageal echocardiography mid-esophageal view (four chamber). TEE: transesophageal echocardiogram

Transthoracic and transesophageal echocardiograms revealed a large mobile echodense structure, 0.7x1.22 cm, with independent motion attached to the anterior leaflet of the tricuspid valve, suggestive of tricuspid vegetation. It also showed mild thickening of the anterior leaflet of the pulmonic valve and possible pulmonic vegetation (Figures 3A-3C). The patient was not deemed a surgical candidate given the vegetation size, no evidence of septic pulmonary emboli, and clinical improvement. The patient's antibiotic regimen was de-escalated to vancomycin to complete the six-week course. Repeat blood cultures were negative, suggesting clearance of bacteremia.

## Discussion

Austrian syndrome is a rare but fatal manifestation of *Streptococcus pneumoniae* bacteremia. It is a triad of streptococcal pneumonia, meningitis, and endocarditis. Austrian syndrome was first comprehensively described by Robert Austrian in 1957 [1]. This syndrome represents a severe manifestation of invasive pneumococcal disease with multiorgan involvement and unique diagnostic and therapeutic challenges. Despite advancements in antimicrobial therapy and critical care, the syndrome remains associated with high morbidity and mortality, especially with delayed diagnosis and incomplete treatment [9]. 

In Austrian syndrome, the lungs usually present as the initial infectious focus, from which *Streptococcus pneumoniae* disseminates hematogenously to the heart valves and central nervous system [10]. The pulmonary component of the syndrome often presents with typical features of community-acquired pneumonia, such as fever, cough, pleuritic chest pain, and dyspnea, along with radiographic findings of lobar consolidation, most frequently lower lobes [11]. In many reported cases, the pulmonary infection precedes the recognition of endocarditis or meningitis by several days, suggesting that pneumonia may act as the entry point for systemic invasion [12]. *S. pneumoniae *endocarditis is uncommon in this post-antibiotic era and accounts for less than 3% of all cases of infective endocarditis. However, when present, it commonly affects the aortic valve and rapidly progresses into severe aortic regurgitation, heart failure, or systemic embolism. This confers greater than 60% mortality without surgical treatment [13]. The mitral valve may also be involved, especially in individuals with pre-existing valvular disease. Echocardiography, especially transesophageal, is essential in the evaluation of suspected endocarditis [14].

The diagnosis of Austrian syndrome is based on a combination of clinical suspicion and microbiologic and radiological evidence. Blood cultures remain the cornerstone of diagnosis, with *S. pneumoniae *isolated in up to 85-90% of cases when all three components are present [9]. Simultaneous or sequential isolation of *S. pneumoniae *from blood, cerebrospinal fluid (CSF), and sputum strongly supports the diagnosis [8]. Echocardiography is essential to confirm endocarditis. Transesophageal echocardiography (TEE) is preferred over transthoracic echocardiography (TTE) due to its superior sensitivity in detecting vegetations, particularly on the aortic valve, which is most commonly affected in Austrian syndrome [15].

The management of Austrian syndrome involves prompt and timely initiation of high-dose intravenous antimicrobial therapy targeting *S. pneumoniae *to achieve effective concentrations in both the central nervous system and the bloodstream. Empiric antimicrobial treatment should cover multidrug-resistant strains. Guidelines recommend broad coverage with high-dose intravenous ceftriaxone or cefotaxime along with vancomycin until susceptibility results are available [16]. Once susceptibilities are available, antibiotics can be de-escalated to monotherapy with high-dose beta-lactam for penicillin-susceptible strains [17]. The duration of therapy depends on the involvement of the endocardium and meninges - typically four to six weeks for endocarditis and 10-14 days for meningitis. In confirmed meningitis, dexamethasone should be administered adjunctively, before or with the first dose of antibiotics, to reduce neurologic complications and mortality [18]. Surgical intervention is often required in cases of significant valvular dysfunction, congestive heart failure, or persistent bacteremia. This has improved survival in pneumococcal endocarditis when indicated [19]. Patients with multisystem involvement also benefit from supportive care, including hemodynamic stabilization, respiratory support, and management of increased intracranial pressure. Despite aggressive therapy, Austrian syndrome carries a high mortality rate, ranging from 30% to 60%, highlighting the importance of early recognition and a coordinated, multidisciplinary treatment approach [20]. The role of pneumococcal vaccination cannot be overstated in preventing invasive pneumococcal disease. The conjugate and polysaccharide vaccines have significantly reduced the incidence of pneumococcal infections across multiple populations. However, vaccine failure and non-vaccine serotypes continue to pose risks, especially in immunocompromised individuals [5,21].

Our patient’s initial presentation was bibasilar pneumonia, which likely served as the primary infectious focus, consistent with reports that pulmonary involvement precedes dissemination to the central nervous system and cardiac valves. This case also highlights several atypical yet clinically important features. While pneumococcal endocarditis most commonly affects the aortic valve, our patient developed tricuspid endocarditis, likely influenced by his history of intravenous drug use and immunosuppressive state from HIV infection. Microbiological and radiographic evidence strengthened the diagnosis and further emphasized the invasive nature of the infection. Our patient received guideline-directed therapy with broad-spectrum coverage before de-escalation based on susceptibilities. Surgical intervention was deferred due to clinical stability and absence of embolic complications. His favorable response underscores the importance of early recognition, multidisciplinary management, and aggressive antimicrobial therapy in improving survival, even in high-risk hosts.

## Conclusions

Austrian syndrome is a rare but life-threatening condition that requires high suspicion for diagnosis and a multidisciplinary approach for management. This case highlights the classic Osler’s triad of *Streptococcus pneumoniae* pneumonia, meningitis, and tricuspid valve endocarditis caused in an immunocompromised individual. The patient’s course was marked by rapid neurologic deterioration necessitating prompt escalation of antimicrobial therapy and extensive diagnostic workup. The diagnosis was confirmed with positive blood and cerebrospinal fluid cultures, as well as echocardiographic evidence of valvular vegetations. The patient had a favorable response to intravenous antibiotic therapy with clearance of bacteremia without any surgical intervention. This case delineates the importance of early diagnosis and multidisciplinary management in Austrian syndrome. Although it is rare in this era of antibiotics and vaccination, physicians must remain aware of this syndrome, especially in high-risk populations, and continue emphasizing preventive strategies, including pneumococcal immunization, especially in the immunocompromised population, to reduce disease incidence.
